# The relationship between parenting behavior and the personality of kindergarten children

**DOI:** 10.3389/fpsyg.2023.1048391

**Published:** 2023-02-22

**Authors:** Johanna Däschle, Carmen Hofmann, Jennifer Wernicke, Ute Ziegenhain, Christian Montag, Markus Kiefer

**Affiliations:** ^1^Section for Cognitive Electrophysiology, Department of Psychiatry and Psychotherapy III, Ulm University, Ulm, Germany; ^2^Transfer Center for Neuroscience and Education (ZNL), Ulm University, Ulm, Germany; ^3^Department of Molecular Psychology, Institute of Psychology and Education, Ulm University, Ulm, Germany; ^4^Department of Child and Adolescent Psychiatry and Psychotherapy, Research House, University Hospital Ulm, Ulm, Germany

**Keywords:** personality, biosocial personality model, parenting behavior, responsive parenting, demanding parenting, kindergarten children

## Abstract

According to Cloninger’s biosocial model of personality touching upon temperament and character, personality development is a lifelong adaptive process that begins in early childhood. Similarly, theories of parenting behavior and attachment predict that associations between personality and parenting behavior should be found in young children. The present study therefore had the goal to investigate, whether associations between parenting behavior and personality in terms of Cloninger’s temperament and character dimensions previously found in adolescence and adults can already be observed in kindergarten children. This study assessed personality in a sample of 324 kindergarten children (169 girls/155 boys) aged 3–6 years (*M*_age_ = 4.59, SD = 0.90). Parents rated their children’s temperament and character using the JTCI 3–6 R questionnaire, which has been specifically developed to measure personality dimensions in three to six-year-olds according to Cloninger’s model. Character traits (especially Self-Transcendence), which reach mature levels in adults, may not be reliably assessed in three-year-old children. Parenting behavior was documented using the DEAPQ-EL-GS self-report questionnaire measuring the parenting behavior dimensions Responsiveness and Demandingness. Correlation analyses revealed that responsive parenting behavior was positively related to the personality dimensions Reward Dependence, Self-Directedness, and Self-Transcendence. Demanding parenting behavior was positively related to the personality dimension Novelty Seeking, but negatively related to the personality dimensions Persistence, Self-Directedness and Cooperativeness. Although the cross-sectional design of our study prevents unequivocal conclusions about the causal direction of these associations, our results highlight possible differential consequences of responsive vs. demanding parenting behavior for personality development in children in line with theories of parenting behavior and attachment. Our results thus advance earlier work in adolescents and adults, by showing that parenting behavior influences the development of the child’s personality according to Cloninger’s biosocial model already in three to six-year-olds.

## 1. Introduction

Personality manifests in rather stable motivational, emotional, cognitive, and behavioral tendencies (Mischel, 1976; Komulainen et al., 2014; Montag and Elhai, 2019), although personality traits may be subjected to plastic changes to some extent (Bleidorn et al., 2018). The investigation of personality development is important as personality not only can be linked to daily decisions and interactions with our environment (Komulainen et al., 2014), but also to other important life outcomes including health behavior (Bogg and Roberts, 2004) and longevity (Jackson et al., 2015). Personality development is shaped from birth by environmental factors and socio-cultural learning processes (Cloninger, 1986; Cloninger et al., 1993, 2019) in interaction with the genetics of a person (Montag et al., 2020). Given the importance of environmental influences on personality development, the present study has the goal to assess the relationship between parenting behavior and the child’s personality in kindergarten children.

Several personality models aim to shed light on the psychobiological basis of personality. A review of all these theories is beyond the scope of this article, but prominent theories include Eysenck’s PEN model (Eysenck, 1991), Gray’s revised reinforcement sensitivity theory (Markett et al., 2014; Reuter et al., 2015), Zuckerman’s sensation seeking theory (Zuckerman and Cloninger, 1996) and more recently also Panksepp’s Affective Neuroscience Theory (Davis and Montag, 2019), which can be applied to personality psychology (Marengo et al., 2021; Montag et al., 2021). The present work focuses on Cloninger’s biosocial model of personality, which is one of the most widely investigated personality models in healthy and clinical populations. Cloninger et al. (1993) postulated a theory-based, biologically, psychologically, and sociologically founded model of personality and combined it with a factor-analytical approach. Cloninger et al.’s (1993) biosocial model is intended to be applied to healthy and clinical populations as well as to individuals in different age ranges. According to this model, personality consists of two components: temperament and character (Cloninger, 1986; Cloninger et al., 1993). Cloninger (1994, p. 266) himself mentioned that “temperament involves individual difference in percept-based habits and skills …, whereas character involves differences in concepts about one’s self in functional relation to parts of the whole field experience”. Thereby, the temperament refers to basic, quasi-automatic reaction tendencies to stimuli. It is considered as the more biologically founded basis of personality, which remains a relatively stable reaction pattern from childhood to old age. Nevertheless, a longitudinal study reported normative changes in some temperament traits in adults assessed in the age range between 20–45 years indicating some developmental malleability (Josefsson et al., 2013a). The character refers to the characteristics of an individual, which express the personal goals and values. The character is assumed to be more dependent on socio-cultural learning processes and includes the cognitive and motivational as well as the learned aspects of personality (Rutter, 1989; Goth and Schmeck, 2009). Furthermore, the character includes the self-concept of the individual (Cloninger et al., 1993). Although theoretically temperament has a stronger genetic basis than character, recent genome-wide association studies revealed a substantial genetic basis for both temperament and character profiles (Cloninger et al., 2019; Zwir et al., 2020, 2021).

Cloninger’s biosocial theory of personality distinguishes four temperament and three character dimensions. The following temperament dimensions are proposed: Novelty Seeking has been defined (against an animal framework) as “frequent exploratory activity, approach to novel stimuli, and active avoidance or skilled escape from aversive stimuli” (Cloninger, 1994, p. 269) as well as “impulsive decision making, extravagance in approach to cues of reward, and quick loss of temper and active avoidance of frustration” (Cloninger et al., 1993, p. 977). Harm Avoidance is manifested in the degree of risk avoidance, shyness, a tendency to worry and physical fatigue. Cloninger (1994, p. 269) characterizes Reward Dependence by “warm social affiliations, distress in response to social separation, and sympathy or sensitivity to social cues”. Persistence represents the level of eagerness to work, joy in challenges and effort, ambition, and the tendency to perfectionism.

The following three character dimensions are assumed: Self-Directedness describes “the ability of an individual to control, regulate, and adapt behavior to fit the situation in accord with individually chosen goals and values” (Cloninger et al., 1993, p. 979). Cooperativeness is defined by the “identification with and acceptance of other people” (Cloninger et al., 1993, p. 980). It comprises, social tolerance, empathy, helpfulness, compassion, and moral principles, rather than hostile revengefulness and selfishness (Cloninger, 1994, p. 270). Finally, Self-Transcendence includes abstract and imaginative connection with man and nature as well as spirituality, creative self-forgetfulness and global thinking (Cloninger et al., 1993).

Although character traits are assumed to reach a mature level only much later in life, they start to develop in childhood (Cloninger, 1994). The Junior Temperament and Character Inventory JTCI 3–6 R (Goth and Schmeck, 2009), the instrument we used in our study, has been specifically developed and validated to measure temperament and character traits of Cloninger’s model in kindergarten children aged of 3–6 years. The JTCI 3–6 R has been constructed to measure the developmental trajectory of temperament and character traits of Cloninger’s model (Goth and Schmeck, 2009). Although one might question how far concepts such as Self-Transcendence can be developed in children, research from Alvarenga et al. (2017) is of relevance, pointing to the idea that children between three and six years show “intuitive-projective faith” (p. 438). Of note, spirituality can also be defined in different ways when doing research in children (going beyond the faith system). Houskamp et al. (2004) speak of “an inner belief system, on which a person relies for strength and comfort” (p. 221).

Environmental factors and socio-cultural learning processes shape personality from birth (Cloninger, 1986; Cloninger et al., 1993, 2019) in interaction with the genetics of a person (Montag et al., 2020). The influence of genetics is best understood by complex gene by environment interactions (e.g., Plomin et al., 1977; Krueger et al., 2008; Distel et al., 2011; Ayoub et al., 2019; Zwir et al., 2020, 2021). Nevertheless, despite the role of genetics, the immediate environment of the individual plays a pivotal role in personality development.

Childhood can be considered as the early stage of personality development. Among others, the caregivers establish the child’s environment. For that reason, parenting behavior can be seen as a crucial environmental factor influencing personality development (e.g., Reti et al., 2002; Ayoub et al., 2019). Parenting behavior includes intentional behavior such as verbal communication as well as non-intentional behavior such as gestures, facial expression or vocal pitch (Krohne and Hock, 2018), although non-verbal behavior can also be shown by parents intentionally in some occasions. At a level more general than concrete parenting behavior, a typology of parenting styles has been proposed, which describes parent–child interactions across a wide range of situations and also includes the affective quality of the parent–child interaction (Darling and Steinberg, 1993).

According to the popular model of parenting styles of Baumrind (1971) and its extension of Maccoby (1992), parenting behavior can be characterized by the two dimensions Responsiveness and Demandingness. The dimension Responsiveness describes the extent to which parents respond to the children’s needs and show them affection and trust. On this dimension, affection, encouragement, recognition, and trust are contrasted with devaluation and rejection. The dimension Demandingness represents the extent to which the child’s activities are controlled and how much behavioral regulation is demanded. The four parenting styles, which can be arranged on these two dimensions are the authoritative parenting style, which is high in responsiveness and high in demandingness, parents with an authoritarian parenting style are demanding but not responsive, permissive parents, in turn, are responsive but not demanding, and the negligent parenting style consists of low responsiveness and low demandingness.

Although many studies did not explicitly classify parenting behavior according to the dimensions Responsiveness and Demandingness (Baumrind, 1971), the variable parental warmth and care can be taken as index for responsive parenting behavior (Parker et al., 1979). The variable overprotection, which manifests itself through high parental control and intrusive, infantile and highly protective behavior (Cavedo and Parker, 1994) and rejection (Parker et al., 1979), and the variable behavioral restrictiveness and denial of psychological autonomy (Reti et al., 2002) can be seen as index for demanding parenting behavior.

Theories in the fields of parenting behavior (Baumrind, 1971) and attachment (Bowlby, 1977) emphasize the importance of the parent–child relationship on children’s emotional and cognitive development including the growth of temperament and character. Parenting behavior is an important factor for establishing the affective quality of the parent–child relationship. Caregivers are thought to initially regulate behavior and emotions of the infant and facilitate the progression from external to internal self-regulation, depending on parental responsiveness to the child and autonomy support (Rothbart et al., 2003, 2011). High parental responsiveness, which is characterized by a warm and supportive family environment, is closely linked to child attachment security (Bowlby, 1977). A secure attachment encourages the child to explore the environment in an autonomous fashion (Ainsworth and Bell, 1970), fosters the practice of self-controlled actions and supports efficient regulation of emotions (Rothbart et al., 2003, 2011). Highly demanding parenting behavior, which is characterized by exertion of parental control and low autonomy of the child, is assumed to increase the child’ emotional distress while reducing the child’s sense of self-efficacy and feeling of self-determination (Baumrind, 2005). Based on these theoretical considerations, parenting behavior is expected to be related to the offspring’s temperament and character traits (see also Cloninger, 1994; Kiff et al., 2011).

Several studies have already investigated the relationship between parenting behavior and the child’s personality development in light of Cloninger’s biosocial personality theory (for a review, see Kiff et al., 2011). Responsive parenting behavior was associated with increased Reward Dependence (Ruchkin et al., 1998; Richter et al., 2000; Reti et al., 2002; Kitamura et al., 2009), Persistence (Ruchkin et al., 1998; Reti et al., 2002; Kitamura et al., 2009; Takeuchi, 2011), Self-Directedness (Ruchkin et al., 1998; Reti et al., 2002; Kitamura et al., 2009; Takeuchi, 2011), and Cooperativeness (Ruchkin et al., 1998; Kitamura et al., 2009). In contrast, responsive parenting behavior was negatively related to Novelty Seeking (Ruchkin et al., 1998; Kitamura et al., 2009; Takeuchi, 2011), and Harm Avoidance (Ruchkin et al., 1998; Richter et al., 2000; Reti et al., 2002; Kitamura et al., 2009).

Demanding parenting behavior was positively associated with Novelty Seeking (Richter et al., 2000; Reti et al., 2002; Kitamura et al., 2009) and Harm Avoidance (Reti et al., 2002; Kitamura and Kishida, 2005; Oshino et al., 2007; Kitamura et al., 2009; Josefsson et al., 2013b). However, demanding parenting behavior was negatively associated with Persistence (Reti et al., 2002; Kitamura et al., 2009; Takeuchi, 2011), Self-Directedness (Reti et al., 2002; Kitamura et al., 2009; Takeuchi, 2011; Josefsson et al., 2013b), and Cooperativeness (Ruchkin et al., 1998; Reti et al., 2002; Kitamura et al., 2009; Takeuchi, 2011; Josefsson et al., 2013b). For the personality dimension Self-Transcendence associations with demanding or responsive parenting behavior were inconsistent across studies (Ruchkin et al., 1998; Kitamura and Kishida, 2005; Oshino et al., 2007; Kitamura et al., 2009; Josefsson et al., 2013b). Possibly, the heterogeneous findings might be due to different sample characteristics or different parenting behavior questionnaires. It should also be noted that cultural differences in self-transcendence (Josefsson et al., 2011) might explain the heterogeneous findings.

Most of the studies described above assessed parenting behavior and personality within a sample of adolescents or adults, who retrospectively evaluated parenting behavior of their parents, when they were a child. This approach has several limitations: Firstly, it is unknown, whether the reported associations between parenting behavior and offspring’s personality traits is already present in childhood. Secondly, this approach is prone to various biases and allows limited conclusions about the characteristics of parenting behavior in early childhood, because adults frequently cannot retrospectively remember this period in a valid fashion (Finkel and McGue, 1993; Nivison et al., 2021). Assessing the association between parenting behavior and the offspring’s personality in childhood is not only methodologically, but also theoretically important, because both parenting and attachment theories outlined above suggest that the parent–child relationship contributes to the children’s personality development already in early childhood starting in infancy (Rothbart et al., 2011).

In the present study, we therefore investigated the relationship between parenting behavior and child’s personality in kindergarten children and their parents. Investigation of young children such as kindergarten children has several advantages. Firstly, parents can report current parenting behavior and must not rely on retrospective memory over many years. Secondly, we can assess whether parenting behavior is related to the child’s personality already at a relatively young age and not only in adolescence or young adulthood as in several earlier studies. In addition to the theoretical implications, the results of the present study could also help to design intervention programs with a focus on parenting.

Parenting behavior was assessed using the German extended edition of the Alabama Parenting Questionnaire (DEAPQ-EL-GS; Reichle and Franiek, 2009). This questionnaire reflects the two parenting behavior dimensions Responsiveness and Demandingness (Baumrind, 1971; Maccoby, 1992) measured by seven scales of individual parenting behaviors (for details, see the methods section). Child’s personality was assessed by parents’ report using the Junior Temperament and Character Inventory (JTCI 3–6 R, Goth and Schmeck, 2009), which is based on Cloninger’s personality model.

Based on previous work and theoretical considerations, it was assumed that responsive parenting behavior has a positive relationship with Reward Dependence, Persistence, Self-Directedness and Cooperativeness. While responsive parenting behavior was expected to be negatively related to Novelty Seeking and Harm Avoidance, demanding parenting behavior should be positively associated with Novelty Seeking and Harm Avoidance. Demanding parenting behavior should be negatively related to Persistence, Self-Directedness, and Cooperativeness. With regard to the personality dimension Self-Transcendence, we had no specific prediction given the inconsistent associations in the literature. In line with theories of parenting behavior (Baumrind, 1971) and attachment (Bowlby, 1977) and in advancing earlier work in adolescents and adults, such associations between parenting behavior and personality dimensions would demonstrate that possible differential consequences of responsive vs. demanding parenting behavior for personality development are already present in children.

## 2. Materials and methods

We analyzed data of the Ulm Gene Brain Behavior Project (UGBBP) data base collected within two substudies (termed substudy 1 and substudy 2).

### 2.1. Participants

The total sample of the study consisted of *n* = 324 kindergarten children (substudy 1: *n* = 203, substudy 2: *n* = 121, 169 females, *M*_age_ = 4.59 years, SD = 0.90 years) aged 3–6 years and their parents. All children and their parents were Caucasian. The average age of the children’s parents was 38.63 years (SD = 4.95 years, range 24–61 years), with mothers averaging 37.29 (SD = 4.90 years) and fathers 39.98 (SD = 6.17 years) years. In years, the school education of both parents consisted of average *M* = 11.70 (SD = 1.41 years) years, whereby mothers and fathers did not differ significantly in their school education (*t*(635) = −0.02, *p* = 1.00). In the following, we provide some information with regard to the different degrees of the German education system typically received after certain years of school education. The first degree of secondary school education is received after 9 years of schooling (minimum of obligatory secondary school education), the second degree is received after 10 years of schooling. The highest secondary school degree qualifying for university admission is received after 12 or 13 years of schooling depending on the German state. The subsamples did not differ in children’s gender distribution or in parent’s age and educational level. There was only a significant difference in the age of the children (substudy 1: Mage = 5.18 years, SD = 0.78 years; substudy 2: Mage = 4.39 years, SD = 1.05 years; *t*(322) = 7.727, *p* < 0.001), which can be attributed to the different age range as inclusion criterion in the two substudies. The sample of the parents and their children under investigation is drawn from a population of educated German Caucasian middleclass families.

We recruited the children and their parents in kindergartens of the cities Ulm, Neu-Ulm (Germany) and of surrounding districts. First, the directors of the kindergartens were asked for consent to recruit children and parents in their institutions. Thereafter, to inform the parents and their children about our study, we distributed flyers in the kindergartens with information about the purpose of the study. If the parents and the children were interested to participate, they were asked to contact the research team *via* email, phone or regular mail. Parents then received further study information, the informed consent form and the questionnaires described below by regular mail. Parents returned the signed informed consent form and the questionnaires to the research team by regular mail. The questionnaires were enclosed in a separate envelope, on which only a code generated by the parents was printed, in order to ensure anonymity of the respondents.

Inclusion criteria were the children’s age of 4–6 years in substudy 1 and of 3–6 years in substudy 2 as well as sufficient German language skills of the parents and the children. Another inclusion criterion was the absence of any known mental or neurological disorder or developmental delay in the child according to parents’ report. A total of 346 children participated in either substudy, 17 had to be excluded due to missing questionnaire data, one child due to developmental delay and one due to exceeding the maximum age. One child was included in both subsamples, so that one data set was removed. Another child had double questionnaire data, so that the questionnaire, which was submitted later, was removed. One child had eight missing items in the JTCI questionnaire and was excluded according to the manual. Children’s legal caregivers gave written informed consent. The ethics committee of Ulm University, Ulm, Germany approved the study protocol (290/15). All procedures were in accordance with the ethical standards of the institutional and/or national research committee and in line with the 2013 Helsinki declaration and its later amendments or comparable ethical standards. There are no conflicts of interests to be declared.

### 2.2. Instruments

For 83.64% (*n* = 271) of the children the questionnaires were completed by the mother, while the fathers completed the questionnaires in only 13.88% (*n* = 45) of the cases and in 2.16% (*n* = 7) of the cases the parents completed the questionnaires together.

#### 2.2.1. Personal data

Using an in-house questionnaire, we collected demographic and familiar data of each child including age, gender, number of biological siblings, mother tongue, and ethnic origin. In addition, the questionnaire contained questions about the parents’ personal data, such as age, highest educational and vocational qualifications.

#### 2.2.2. Personality

The Junior Temperament and Character Inventory (JTCI 3–6 R; Goth and Schmeck, 2009) was used to measure the child’s personality and is based on Cloninger’s biosocial model of personality. The JTCI 3–6 R administered in this study was designed for the age group 3–6 years. This instrument has been explicitly constructed and validated to measure the developmental trajectory of temperament and character traits of Cloninger’s model. The JTCI 3-6R according to the German manual being published at Hogrefe (Goth and Schmeck, 2009) shows sufficient psychometric properties (see also information on internal consistencies touching upon the present data below). Validity of the JTCI 3–6 R was demonstrated based on associations with related personality constructs as well as with regard to associations with corresponding psychopathology (Goth and Schmeck, 2009; d’Huart et al., 2022). The inventory is a parent report and contains 86 items that are answered on a five-point-Likert-scale (0 = no, 1 = rather no, 2 = partly/partly, 3 = rather yes, 4 = yes). It measures the seven personality dimensions described in detail above: the four temperament dimensions Novelty Seeking, Harm Avoidance, Reward Dependence, Persistence, and the three character dimensions Self-Directedness, Cooperativeness and Self-Transcendence. All items of the JTCI 3-6R questionnaire, including those of the scales measuring character traits, which are assumed to reach a mature level in adulthood, have been worded to capture personality dimensions of Cloninger’s model in children. The scale of Self-Directedness assesses a little child’s tendency to initiate and regulate actions based on the own intention. The scale of Cooperativeness refers to the child’s tendency to show respectful behavior, when interacting with others, and to accept the will of other people. The scale of Self-Transcendence measures the child’s tendency to be engaged in imaginative thoughts (e.g., being a character from a movie or book) and to be concerned with issues associated with life, death, myths or religious beliefs. We calculated the scales as mean values according to the manual and under the rules for treatment of missing data noted there. A higher value on a scale means a higher expression of the personality dimension. Internal consistencies (Cronbach’s alpha) ranged from *α* = 0.70 (RD) to *α* = 0.88 (C) here. There was no reason to remove items to increase reliability. The assumed factor structure of the model was proven by explorative and confirmatory factor analyses in an earlier study (Goth and Schmeck, 2009).

#### 2.2.3. Parenting behavior

Parenting behavior was surveyed using a modified, extended German version of the Alabama Parenting Questionnaire (DEAPQ-EL-GS; Reichle and Franiek, 2009). In its original version, the self-assessment questionnaire consists of 40 items, which are answered on a five-point-Likert-scale (1 = almost never, 2 = rarely, 3 = sometimes, 4 = often, 5 = almost always). The original version of the questionnaire was designed for parents of primary school children. To capture the age group of three to six-year-old children, items that seemed inadequate for kindergarten children were removed (see Supplementary Table S1), following Schreyer-Mehlhop and Petermann (2011). The modified version consisted of 35 items in total. The DEAPQ-EL-GS reflects the two dimensions responsiveness and demandingness (Baumrind, 1971; Maccoby, 1992) measured in seven subscales of parenting behaviors. The subscale positive parenting describes a warm, friendly, and child-centered approach, while responsible parenting is characterized by a sense of responsibility and well-considered, non-impulsive actions. Involvement describes the active parental contribution to the child’s development through participation in the activities of the child. The lack of information of parents about the activities and social contacts of the child characterizes poor monitoring. Inconsistent discipline refers to parenting behavior characterized by incongruences or changes of announced and actual imposed consequences in response to the child’s behavior. Powerful assertion is characterized by parental coercion, control and negative emotional mood. Corporal punishment involves the use of corporal punishment to regulate the child’s behavior. Average values across the items of each of the parenting behavior scales were calculated. Higher values indicate a higher expression of the respective parenting behavior. The internal consistency of the scales was determined using Cronbach’s *α* and Spearman-Brown statistics. The internal consistence (Cronbach’s alpha) of the subscales calculated in this study were in a range of *α* = 0.23 (poor monitoring) and *α* = 0.76 (inconsistent discipline). The poor monitoring scale showed low internal consistency (Cronbach’s *α* = 0.23; Spearman-Brown value = 0.22) due to the removal of four items, which were not adequate for preschool children (see Supplementary Table S1). In this study, the poor monitoring scale consisted of only two items. The scale involvement contained one item less (see Supplementary Table S1). All the other scales had the original number of items (Reichle and Franiek, 2009). We retained the poor monitoring scale in the analyses despite its poor internal consistence, because it addresses an important aspect of parenting behavior included in earlier work. The two parenting behavior dimensions responsiveness and demandingness were calculated from the subscales analogous to the procedure in Reichle and Franiek (2009). For the dimension responsiveness, the averaged sum scores of the subscales positive parenting, responsible parenting, involvement and the inverted scores of the scale poor monitoring were calculated. The parenting dimension demandingness was calculated from the averaged sum scores of the subscales powerful assertion, inconsistent discipline and corporal punishment. A higher value indicates a stronger expression on the respective scale.

### 2.3. Statistical analysis

Statistical analyses were performed using R 3.6.1 (R Core Team, 2019) and the R packages readxl and psych. Alpha level of statistical significance was set at *p* < 0.05 for all analyses. First, we calculated means and standard deviations for all variables. In addition, correlations between personality dimensions and parenting behavior dimensions (responsiveness and demandingness) were calculated. The correlations between personality dimensions and parenting behavior dimensions were controlled for child’s age and gender as well as for parental education by calculating partial correlations. To control results of the correlation analyses for false discovery rate (FDR), the Benjamini and Hochberg (1995) correction was applied. In case of the JTCI 3–6 R missing data were treated according to the manual. In the cases, which had less than five missing values in the JTCI 3–6 R, the main dimensions and mean values were formed according to the manual, in which the missing items were excluded from the scale formation and the mean value was corrected by the number of missing items. Cases with more than five missing values in the JTCI 3–6 R were excluded from analyses (Goth and Schmeck, 2009).

## 3. Results

In the following, descriptive statistics of the questionnaires and the correlative relationships between personality dimensions and parenting behavior dimensions are presented. Table 1 shows the descriptive statistics of the personality and parenting behavior variables. Higher values indicate a higher expression of the respective personality or parenting behavior dimension. An analysis with regard to gender differences in personality can be found in the Supplementary Table S2. In brief, there was only a statistically significant difference in Reward Dependence: Girls showed a higher expression of Reward Dependence than boys.

**Table 1:** Descriptive statistics of personality and parenting behavior.

	*n*	*M*	SD	Median	Min	Max
**JTCI 3–6 R**						
Novelty Seeking	324	1.55	0.66	1.48	0.22	3.54
Harm Avoidance	324	1.22	0.57	1.16	0.00	3.15
Reward Dependence	324	2.53	0.47	2.50	0.92	3.92
Persistence	324	2.51	0.61	2.54	0.86	3.93
Self-Directedness	324	3.1	0.56	3.19	0.96	4
Cooperativeness	324	2.66	0.72	2.71	0.17	4
Self-Transcendence	324	2.2	0.67	2.19	0.29	4
**DEAPQ-EL-GS**						
Responsiveness	319	1.92	0.27	1.94	1.06	2.54
Demandingness	310	2.62	0.34	2.64	1.53	3.72

JTCI 3–6 R Range: 0–4; DEAPQ-EL-GS Range: 1–5.

The correlations between the two dimensions of parenting behavior and the child’s personality dimensions are presented in Table 2. The parenting behavior dimension Responsiveness showed significant positive correlations with the personality dimensions Reward Dependence, Self-Directedness and Self-Transcendence. The parenting behavior dimension Demandingness had a significant positive association with the personality dimension Novelty Seeking, and significant negative associations with the personality dimensions Persistence, Self-Directedness and Cooperativeness. Other correlations were not significant.

**Table 2:** Correlations between personality dimensions and parenting behavior.

	NS	HA	RD	*P*	SD	*C*	ST
res	−0.09	−0.11	0.19**	0.11	0.15*	0.10	0.13*
dem	0.35**	0.06	0.10	−0.13*	−0.15*	−0.29**	0.04

All statistical tests are two-tailed. Reported are partial correlations controlling for age and gender of the child as well as parental education. Statistical significance was adjusted according to the Benjamini-Hochberg correction to control false discovery rate (FDR) (Benjamini and Hochberg, 1995). Asterisks indicate the corrected level of significance: **p ≤ 0.01, and *p ≤ 0.05. res, Responsiveness; dem, Demandingness; NS, Novelty Seeking; HA, Harm Avoidance; RD, Reward Dependence; P, Persistence; SD, Self-Directedness; C, Cooperativeness; ST, Self-Transcendence.

## 4. Discussion

The aim of the present study was the investigation of the relationship between parenting behavior and the personality of children in the kindergarten age. The associations between dimensions of parenting behavior and child’s personality were similar to earlier studies in adolescence and adults (Ruchkin et al., 1998; Richter et al., 2000; Reti et al., 2002; Kitamura and Kishida, 2005; Oshino et al., 2007; Kitamura et al., 2009; Takeuchi, 2011). Our study thus shows that correlational patterns between parenting behavior and offspring’s personality traits as measured with questionnaires in adolescents and adults can also be found in kindergarten children aged between three and six years. It must remain open whether the questionnaires measure the intended personality traits, in particular when the same questionnaires were administered to individuals in different cultures or age groups. Despite these difficulties associated with the appropriate assessment of personality traits in different age and cultural populations, we found a correlational pattern in young children, which was quite comparable to that in adults and adolescents. This suggests that relations between parenting behavior and offspring’s personality traits, which have been found in older individuals, can already be observed in kindergarten children.

Responsive parenting behavior was positively related to the personality dimensions Reward Dependence, Self-Directedness and Self-Transcendence. These results are consistent with previous findings (Ruchkin et al., 1998; Richter et al., 2000; Reti et al., 2002; Kitamura and Kishida, 2005; Oshino et al., 2007; Kitamura et al., 2009; Takeuchi, 2011). It thus seems that a caring, consistent and supporting framework provided by the parents may help children to develop into persons seeking warm affiliations (but also fearing social separation). Moreover, these children tend to better control their behavior based upon personal goals (see also, Rothbart et al., 2011) and more likely experience the spiritual aspects of the self, hence being part of a “unitive whole” (Cloninger, 1994, p. 269). In contrast to the present results, several studies did not observe a significant association of Self-Transcendence with parenting behavior (Ruchkin et al., 1998; Oshino et al., 2007; Takeuchi, 2011; Josefsson et al., 2013b). Possibly, methodological difference between studies such as questionnaires used to assess parenting behavior, the age or cultural background of the offspring under investigation may account for these discrepant findings.

Demanding parenting behavior was positively associated with Novelty Seeking, but negatively associated with Persistence, Self-Directedness and Cooperativeness. These results were in accord with previous studies (Ruchkin et al., 1998; Richter et al., 2000; Reti et al., 2002; Kitamura and Kishida, 2005; Oshino et al., 2007; Kitamura et al., 2009; Takeuchi, 2011). This suggests that children, who received high control from their parents, showed increased explorative activity, but also impulsive decision making and quick loss of temper (Cloninger et al., 1993, p. 977). These children also exhibit a lack of ambition, reduced ability of willed action control based upon personal goals (see also Schneider-Hassloff et al., 2016) as well as decreased acceptance of others and increased selfishness (Cloninger, 1994, p. 270).

Although our observed associations were generally in line with earlier work in adolescence and young adults (Ruchkin et al., 1998; Richter et al., 2000; Reti et al., 2002; Kitamura and Kishida, 2005; Oshino et al., 2007; Kitamura et al., 2009; Takeuchi, 2011), not all expected associations turned out to be significant. The personality dimension Harm Avoidance was not related to any dimension of parenting behavior. Furthermore, contrary to our expectations and earlier work, the personality dimension Novelty Seeking was not significantly negatively associated with responsive parenting behavior, although such an association was obtained at the descriptive level. The failure to find these expected associations in our sample of young children is difficult to explain: It might indicate that these associations between offspring’s personality and parenting behavior emerge later in personality development. Alternatively, it might reflect the somewhat lower statistical power of the present study due to the smaller sample size compared to earlier studies in adolescents and adults.

The correlations between individual personality dimensions and parenting behavior were weak (Self Transcendence) to moderate (Novelty Seeking). This is not surprising, because other factors than parenting behavior are important for child’s personality development. Genetic influences, which were not considered here, have a major influence on both temperament and character profiles (Cloninger et al., 2019; Zwir et al., 2020, 2021). Other familial and psychosocial factors (for example number of siblings, personality of parents, cultural background, etc.) could also contribute to personality development. Nevertheless, the results of this study show that several associations between parenting behavior and the offspring’s personality, as previously observed in adolescent and adult samples (see Richter et al., 2000; Reti et al., 2002; Takeuchi, 2011), can already be seen in kindergarten children.

When interpreting the present results, limitations in methodology and study design must also be taken into account. Parenting behavior was measured with the DEAPQ-EL-GS (Reichle and Franiek, 2009). This questionnaire showed low reliability in the poor monitoring scale, because of the reduction of items to adjust the questionnaire to the age range of the sample. Although the JTCI 3–6 R questionnaire (Goth and Schmeck, 2009) has been specifically developed to measure personality dimensions according to Cloninger’s model in three to six-year-old children, character traits (especially Self-Transcendence), which reach mature levels in adults, may not be reliably assessed in three-year-old children.

Self-report questionnaires are susceptible for bias due to the tendency of parents to answer socially desirable, because parenting behavior is a topic full of conventions and norm in the society. Related to this issue, assessment of parenting behavior and the child’s personality *via* questionnaire in the same person (here caregiver) could induce common method variance that is “variance that is attributable to the measurement method rather than to the constructs the measures represent” (Podsakoff et al., 2003, p. 879). However, as proposed previously as a remedy (Chang et al., 2010), biases stemming from common method variance are reduced when the questionnaires use a different scale as it was the case in the DEAPQ-EL-GS (Reichle and Franiek, 2009) and in the JTCI 3–6 R (Goth and Schmeck, 2009). As a further remedy (Chang et al., 2010), participants were assured of the anonymity and confidentiality of the study, that there are no right or wrong answers, and that they should answer as honestly as possible. Nevertheless, despite these remedies, it would be desirable to replicate the present results using observational methods to assess parenting behavior independently from the child’ personality (see for instance, Schneider-Hassloff et al., 2016).

Furthermore, assessments of the own parenting behavior and child’s personality depend on the own subjective perception of the person making the assessment. This is suggested by the study of Kitamura et al. (2009), in which there was relatively low agreement (range from *r* = 0.28 to *r* = 0.41) between the self-report on the own parenting behavior and the external report of the spouse. Spouses assessed the parenting behavior of the same person in different ways. The questionnaires in the present study were answered by the mother in 83.64% of the cases and thus mainly recorded maternal parenting behavior. Therefore, it is possible that the reports depended on the gender of the parent. As only a few fathers were included in the present study, we could not calculate parenting behavior separately for mothers and fathers. Due to the cross-sectional study design, all relationships are purely correlative and no causal interpretation is possible. While parenting behavior might shape the child’ personality, it is also conceivable that parents adjust their parenting behavior to the personality characteristics of their child (Ayoub et al., 2019). Furthermore, other variables (e.g., number of siblings, parental personality, etc.) may influence the child’s personality, which were not considered. Finally, sample size of the present study, which was constrained by the number of data sets available in our database, might have been too small to yield sufficient statistical power to detect tiny associations. The sample also mainly consisted of parents and children from educated German Caucasian middleclass families. Future research should therefore further elucidate the relationship between parenting behavior and child’s personality using a longitudinal study design with several measurement points including an even larger, and more heterogeneous sample as the present one.

## 5. Conclusion

In conclusion, the present study provides evidence for associations between parenting behavior and children’s personality according to Cloninger et al.’s (1993) biosocial model in kindergarten children aged between three and six years. Responsive parenting behavior was positively related to Reward Dependence, Self-Directedness, and Self-Transcendence. Demanding parenting behavior was positively related to Novelty Seeking, but negatively related to Persistence, Self-Directedness and Self-Transcendence. Hence, parental behavior characterized by guidance, respect of the child’s autonomy, as well as by a warm approach with care about the child’ activities was related to a socially oriented, cooperative, and self-determined personality of the child. Parental behavior characterized by imposing inconsistent consequences to the child, corporal punishment and little care about the child’s activities was associated with more impulsive, but less ambitious and cooperative personality traits of the child. The direction of these associations between parenting behavior and child’s personality was comparable to earlier studies in adolescence and adults (see Ruchkin et al., 1998; Reti et al., 2002; Takeuchi, 2011). Although the cross-sectional design of our study prevents unequivocal conclusions about the causal direction of these associations, our results suggest that parenting behavior influences the development of the child’s temperament and character already in young children aged between three and six years. In line with theories of parenting behavior (Baumrind, 1971) and attachment (Bowlby, 1977) and in advancing earlier work in adolescents and adults, our results highlight possible differential consequences of responsive vs. demanding parenting behavior for personality development already in young children. The results of the present study could thus help to design intervention programs with a focus on parenting.

## Data availability statement

The raw data supporting the conclusions of this article will be made available by the authors, without undue reservation.

## Ethics statement

The studies involving human participants were reviewed and approved by ethics committee of Ulm University, Ulm, Germany. Written informed consent to participate in this study was provided by the participants’ legal guardian/next of kin.

## Author contributions

CH, JW, UZ, CM, and MK conceptualized the study and contributed to the design of the study. CH and JW conducted the investigation and responsible for project administration. JD performed data curation, formal data analyses, and wrote the original draft of the manuscript. MK and CM supervised the project. All authors reviewed and edited the manuscript and approved the submitted version.

## Conflict of interest

The authors declare that the research was conducted in the absence of any commercial or financial relationships that could be construed as a potential conflict of interest.

## Publisher’s note

All claims expressed in this article are solely those of the authors and do not necessarily represent those of their affiliated organizations, or those of the publisher, the editors and the reviewers. Any product that may be evaluated in this article, or claim that may be made by its manufacturer, is not guaranteed or endorsed by the publisher.
