# Supplementary material for: The relationship between parenting behavior and the personality of kindergarten children
Source: Front Psychol. 2023 Feb 22;14:1048391. doi: 10.3389/fpsyg.2023.1048391 (PMC9992217; doi:10.3389/fpsyg.2023.1048391)
Supplement: Supplementary file 1 [file Table_1.docx]

Supplementary Material

# Table S1: Modified DEAQP-EL-GS (removed Items)

Scale *involvement*:

You are helping your child with homework.

Scale *poor monitoring:*

Your child is out of the house and you do not know exactly where he/she is.

Your child is staying out later in the evening than he or she should.

Your child is out with friends you don’t know.

Your child goes out without an agreed-upon time to be back.
