# Supplementary material for: The relationship between parenting behavior and the personality of kindergarten children
Source: Front Psychol. 2023 Feb 22;14:1048391. doi: 10.3389/fpsyg.2023.1048391 (PMC9992217; doi:10.3389/fpsyg.2023.1048391)
Supplement: Supplementary file 2 [file Table_2.docx]

Supplementary Material

# Table S2: Gender differences in temperament and character according to the JTCI 3-6 R

| *Gender differences in JTCI 3-6 R* | | | | | | | | |  |
| --- | --- | --- | --- | --- | --- | --- | --- | --- | --- |
|  | | male | | | female | | | |  |
|  | *n* | | *M* | *SD* | | *n* | *M* | *SD* | *t-test* |
| *JTCI 3-6 R* |  | |  |  | |  |  |  |  |
| Novelty Seeking | 169 | | 1.62 | 0.67 | | 155 | 1.48 | 0.64 | *t*(322) = 2, *p* = 0.060 |
| Harm Avoidance | 169 | | 1.19 | 0.56 | | 155 | 1.25 | 0.57 | *t*(322) = -0.9, *p* = 0.40 |
| Reward Dependence | 169 | | 2.45 | 0.45 | | 155 | 2.62 | 0.49 | *t*(322) = -3, *p* ≤ .001 |
| Persistence | 169 | | 2.50 | 0.60 | | 155 | 2.53 | 0.63 | *t*(322) = -0.5, *p* = 0.60 |
| Self-Directedness | 169 | | 3.06 | 0.57 | | 155 | 3.15 | 0.55 | *t*(322) = -1, *p* = 0.20 |
| Cooperativeness | 169 | | 2.69 | 0.72 | | 155 | 2.62 | 0.73 | *t*(322) = 0.8, *p* = 0.40 |
| Self-Transcendence | 169 | | 2.14 | 0.66 | | 155 | 2.26 | 0.68 | *t*(322) = -2, *p* = 0.09 |

*Note:* JTCI 3-6 R Range: 0-4

Table S1 shows the mean values in children’s personality dimensions, separately for male and female gender. No differences were found in the personality between boys and girls except for Reward Dependence. Girls showed a statistically significant higher expression of Reward Dependence than boys.
